# Ranging Behaviour of Commercial Free-Range Broiler Chickens 1: Factors Related to Flock Variability

**DOI:** 10.3390/ani7070054

**Published:** 2017-07-20

**Authors:** Peta S. Taylor, Paul H. Hemsworth, Peter J. Groves, Sabine G. Gebhardt-Henrich, Jean-Loup Rault

**Affiliations:** 1 Animal Welfare Science Centre, Faculty of Veterinary and Agricultural Sciences, University of Melbourne, Parkville, VIC 3010, Australia; phh@unimelb.edu.au (P.H.H.); raultj@unimelb.edu.au (J.-L.R.); 2 Poultry Research Foundation, School of Veterinary Science, Faculty of Science, The University of Sydney, Camden, NSW 2570, Australia; peter.groves@sydney.edu.au; 3 Research Centre for Proper Housing, Poultry and Rabbits (ZTHZ), Division of Animal Welfare, University of Bern, Zollikofen, CH-3052, Switzerland; sabine.gebhardt@vetsuisse.unibe.ch

**Keywords:** poultry, pasture, outdoor, range, meat chicken, welfare, Radio Frequency Identification (RFID)

## Abstract

**Simple Summary:**

Free-range chicken meat consumption has increased. However, little is known about how meat chickens use the outdoor range. Understanding ranging behaviour could help improve management and shed and range design to ensure optimal ranging opportunities. We tracked 1200 individual broiler chickens in four mixed sex flocks on one commercial farm across two seasons. More chickens accessed the range in summer than winter. Chickens that accessed the range in winter did so less frequently and for a shorter period of time daily than chickens ranging in summer. The number of chickens ranging and the frequency and duration of range visits increased over the first two weeks of range access and stabilised thereafter. More chickens entered and exited the range through particular doors in the shed. More chickens ranged in the morning and evening compared to the middle of the day. Ranging behaviour decreased with increased rainfall and shed dew point. This study provides knowledge regarding ranging behaviour in commercial conditions that may guide improvements on farm to provide chickens with optimal ranging opportunities.

**Abstract:**

Little is known about the ranging behaviour of chickens. Understanding ranging behaviour is required to improve management and shed and range design to ensure optimal ranging opportunities. Using Radio Frequency Identification technology, we tracked 300 individual broiler chickens in each of four mixed sex ROSS 308 flocks on one commercial farm across two seasons. Ranging behaviour was tracked from the first day of range access (21 days of age) until 35 days of age in winter and 44 days of age in summer. Range use was higher than previously reported from scan sampling studies. More chickens accessed the range in summer (81%) than winter (32%; *p* < 0.05). On average, daily frequency and duration of range use was greater in summer flocks (4.4 ± 0.1 visits for a total of 26.3 ± 0.8 min/day) than winter flocks (3.2 ± 0.2 visits for a total of 7.9 ± 1.0 min/day). Seasonal differences were only marginally explained by weather conditions and may reflect the reduction in range exposure between seasons (number of days, hours per day, and time of day). Specific times of the day (*p* < 0.01) and pop-holes were favoured (*p* < 0.05). We provide evidence of relationships between ranging and external factors that may explain ranging preferences.

## 1. Introduction

Free-range chicken meat consumption has increased in some countries [1,2] largely driven by consumer perception that free-range housing is more natural and better for chicken welfare [3]. However, there is little scientific knowledge regarding how the broiler chickens themselves perceive and utilise the outdoor range, despite access to an outdoor range being a unique feature of free-range housing systems. Theoretically, providing access to an outdoor range provides animals with some control to choose when, where, and how to spend their time. Monitoring these choices can permit an understanding of what free-range broiler chickens want, which is an integral part of defining and safeguarding welfare [4]. Chickens may access the outdoor range as the range area provides opportunities to explore a more complex environment than the typical indoor shed environment. However, chickens may also access the range to avoid negative stimuli, such as experiences in the shed that may be uncomfortable, frightening, or painful. Hence, monitoring broiler chicken ranging behaviour and modulations in response to environmental factors can provide insights into the factors underlying broiler chickens’ motivation to range.

Accessing the range may depend on external stimuli such as weather conditions and range microhabitats. Indeed, previous studies indicate that broiler chicken ranging behaviour is affected by time of day, weather variables (rainfall, direct sunlight, temperature, and wind speed), and resources on the range (e.g., trees and straw huts) [5,6,7,8]. However, how such parameters affect ranging patterns of individual broiler chickens, in terms of frequency and duration of range visits have not been reported.

Historically, research suggests that broiler chicken range use is low, reporting that only 3 to 27% of a flock will access the range [5,6,7,9,10]. Such investigations into ranging behaviour utilised scan sampling methods, counting the number of chickens on the range area throughout the day at particular points in time [5,6,7,9,10]. This methodology may underestimate flock range use, as different chickens may access the range at different times in the day. Indeed, recent investigations monitoring individual broiler chicken ranging behaviour suggests that range use is higher than previously reported investigations using scan sampling methods, reporting in the order of 75 to 95% of chickens in a flock accessing the range [11,12]. Therefore, range use is greater than previously thought when assessment is made at the individual level rather than at the flock level.

Understanding ranging behaviour of broiler chickens in response to environmental factors is necessary to develop management practices and shed and range designs that optimise opportunities to range. With this focus, we tracked the individual ranging behaviour of 1200 commercial broiler chickens across four mixed sex flocks on one commercial farm in two seasons to examine the relationships between ranging behaviour and weather and shed conditions, age, and availability of access to the range in terms of hours per day, number of days, and time of day.

## 2. Materials and Methods

### 2.1. Animals and Husbandry

All animals used in this study were approved by the University of Melbourne Animal Ethics Committee (Approval Number 1413428.3).

Four flocks (A–D) of ROSS 308 broiler chickens were studied across two seasonal replicates on one commercial farm in Victoria, Australia: Austral winter (flocks A and B) and summer (flocks C and D). All sheds had chickens from the same hatchery, same feed, same manager, and comparable management practices. Placement of the chicks was made on the same day for winter flocks, and four days apart for summer flocks, with placement day counted as day 0. Each flock contained approximately 6000 (Flocks A and C) or 10,000 (Flocks B and D) broiler chickens kept at a maximum indoor stocking density of 34 kg/m^2^, maintained by removing (“thinning”) approximately 35% of the flock (chosen based on their location in the shed) for slaughter around 35 days of age, described hereafter as “partial depopulation”. The second, and final pick up occurred at 49 days of age and removed all remaining chickens for slaughter, described hereafter as “complete depopulation”. Shed one (flocks A and C) measured 40.5 m × 9.3 m and shed two (flocks B and D) 50.5 m × 12.3 m. The sheds were mechanically ventilated with fans. Natural ventilation was provided when automatic curtains were lowered 1 to 2 m on the side walls of the shed stopping 1 m above the shed floor. The shed wall was solid from the ground to 1m above, therefore even when the curtains were fully opened chickens could not see the range area except through opened pop-holes. Curtains opened automatically based on shed temperature and humidity and thus varied daily. Brooding occurred in the back half of the shed and was temperature controlled by gas heaters. Feed and water were provided ad libitum inside the shed, but never in range areas. Light (20 to 25 lux) was provided on a 23:1 L:D cycle from 0 to 7 days of age and 16:8 cycle until complete depopulation, excluding the three days prior to partial and complete depopulation when light cycle was 20:4. Two tiered perches were provided in each shed (2.7 m/1000 birds) and plastic red chains were hung (20 cm) from drinker lines spread evenly throughout the shed. In winter flocks, management “turned the litter” during ranging hours on the eighth day of range access. “Turning the litter” was achieved by rotary hoeing the litter throughout the entire shed.

### 2.2. Study Site

**Figure 1:**
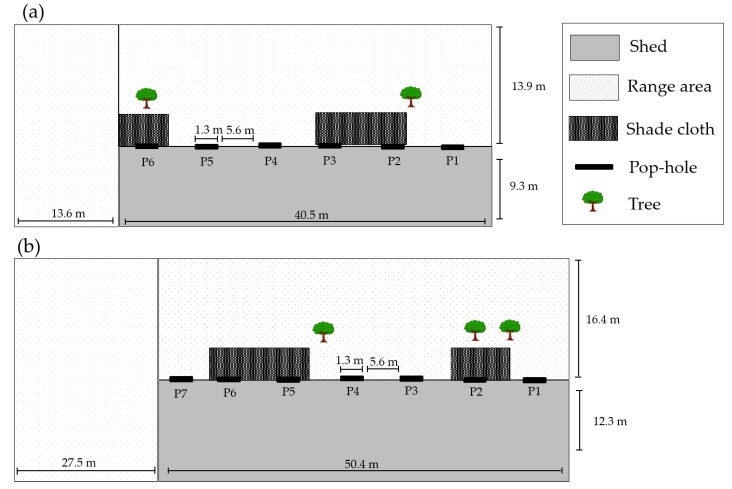
Diagram of study sheds and range areas (**a**) shed one, flocks A and C and (**b**) shed two, flocks B and D. Pop-holes are numbered (P1–P7) sequentially from the front of the shed (shed access point).

Each shed had access to an outdoor range (54.1 × 13.9 m and 77.9 × 16.4 m adjacent to the shed wall and 13.6 × 9.3 m and 27.5 × 12.3 m at the back of the shed, for shed one and two respectively; Figure 1) opening to the west in shed one and north in shed two. The maximum outdoor range stocking density was 6.8 birds/m^2^ and 6.2 birds/m^2^ in shed 1 and 2 respectively. The range was accessible through manually operated 1.3 × 0.4 m doors, described hereafter as “pop-holes”, spaced 5.65 m apart, with six pop-holes for shed one (1.3 cm/bird) and seven pop-holes for shed two (0.9 cm/bird). Pop-holes were manually operated by farm staff, and the time of day that pop-holes were opened and closed was dependent on weather conditions at the discretion of farm staff. Restriction of range access by farm staff was not dictated by one parameter (e.g., temperature), but often a combination of various parameters (e.g., low temperature, high rain fall, and fast wind speed). Data regarding reasons for range restriction was not collected, but such management practices are typical of Australian commercial free-range production. Chickens were first provided with access to the outdoor range at 21 days of age: according to the Free Range Egg and Poultry Australian accreditation standards, chickens must be fully feathered before range access can be provided [13]. Providing first access to the range at 21 days is typical of Australian industry practice. Both range areas were fenced; the back of the range was approximately 16 m from an adjacent road for shed one, and the back of another poultry shed in shed two. Two rectangle shade cloth artificial cover structures, 7 to 10 m in length, ran adjacent to the shed wall 3 m into the range and 3 m above three pop-holes in each shed. The range area for shed one was flat. The range area for shed two had an approximate 45° slope starting 7.5 m from the shed wall. Two trees in the shed one range area and three trees in the shed two range area (2 to 3 m high) were present 3 to 4 m from the shed in each range, located behind the artificial shade cloth. Both ranges were covered with grass, starting 1 to 2 m from the shed wall, kept 10 to 20 cm long throughout periods when the range was available. Anecdotally, no visible degradation of grass > 1 to 2 m from the shed was observed during ranging periods.

### 2.3. Individual Tracking

Individual range use was tracked by the Gantner Pigeon RFID System (2015 Gantner Pigeon Systems GmbH, Benzing, Schruns, Austria), with a bespoke program Chicken Tracker that was developed for the use of tracking chickens and previously validated to track laying hens on commercial farms [14,15]. Chickens (*n* = 300/flock) were randomly selected from ten areas evenly spread within the shed; locations varied according to length and width of the shed and distance from pop-holes. Chickens were fitted with a silicone leg band that automatically loosened with leg growth (Shanghai Ever Trend Enterprise, Shanghai, China). Each leg band contained a unique ID microchip (Ø4.0/34.0 mm Hitag S 2048 bits, 125 kHz) that registered as the chickens walked over antennas. Leg bands were put on three to four days before range access was first provided to allow chickens to habituate to them. Antennas were attached to both sides of each pop-hole (i.e., indoor and outdoor) to determine the direction of movement by each tagged chicken and thus calculate the frequency and duration of range use. Antennas were placed prior to placement of chicks. Chickens were marked with blue or green stock paint (FIL Tell Tail, GEA, New Zealand) on tail and wing feathers to identify tagged chickens in order to retrieve leg bands at the end of the study. In winter flocks, chickens were tracked from the first day that range access was permitted (21 days of age) for 10 days prior to partial depopulation (30 to 33 days of age). Due to logistical reasons, tracking chickens until complete depopulation was prevented. Chickens in summer flocks were tracked daily from the first day that range access was permitted (21 days of age) for 24 days (flock C) and 21 days (flock D) prior to complete depopulation (43 to 45 days of age). No tagged chickens were removed from the flock during partial depopulation in summer flocks. Chickens were excluded from analysis if tags were not recovered or functional at the end of the trial.

### 2.4. Weather Conditions

Weather variables were recorded every 10 min on site in summer via a weather station (Ambient Weather, Chandler, AZ, USA). However, due to equipment failure, weather variables were collected twice daily in winter by the Bureau of Meteorology weather station located 20 km from the farm site [16]. Climate data loggers were placed inside each shed during the summer replicate and recorded temperature, humidity, and dew point every ten minutes. Climate data loggers were not available for winter flocks.

### 2.5. Statistical Analysis

RFID data were cleaned with SAS^TM^ (v 9.3, SAS institute Inc., Cary, NC, USA) using a modified macro [14]. All range visits <10 s were treated as false positives and removed from analysis.

Descriptive ranging data were generated using MATLABTM and Statistics Toolbox Release R2016b (The MathWorks Inc., Natick, MA, USA). Statistical analysis was performed with SPSS statistical software (v 22, IBM Corp, Armonk, NY, USA). Non-ranging chickens were excluded from the analysis that investigated the frequency and duration of range use. In winter flocks, the eighth day of ranging data was analysed separately, due to management turning the litter on that day.

Spearman’s rho correlation coefficients were utilised to examine the relationship between latency to access the range and frequency and duration of range use and duration per range visit. As no random variables could be controlled for in non-parametric correlation analysis, analyses were conducted on individual flock data. All other data met the criteria of normality and homogeneity of variance and therefore parametric statistical tests were used. Pearson’s partial correlation coefficients were used to examine relationships between cumulative ranging day (relative to first availability) and the frequency and duration of range use and the number of chickens that accessed the range, controlling for flock. Pearson’s partial correlation coefficients were also used to identify relationships between the number of hours the range was available daily and the number of chickens on the range, the daily mean duration of a range visit, and the daily frequency and duration of range visits, controlling for flock and age.

Linear regression models were constructed to investigate the chicken and weather variables that predicted the number of chickens that accessed the range. Independent weather variables were included in the analysis if they were correlated with the number of chickens on the range (*p* ≤ 0.10). A variable was removed from the model if it was strongly correlated with another (*r* ≥ 0.70). A maximum of seven variables were included in one model, based on the aforementioned correlation analysis. All possible models were run and the final model included the variables that resulted in the best fit, determined by adjusted r^2^ comparisons and *p*-values indicating significant change in F statistic from a forward stepwise regression analysis. The most parsimonious models are reported with statistically useful variables in the model. Analysis was performed on hourly weather data in summer flocks, but on daily weather data in winter flocks due to technical problems with the onsite weather station in winter (see Section 2.4).

Analysis of Variance (ANOVA) models were used to investigate the use of each pop-hole in each shed for range entry and exit and the number of ranging chickens between seasons and flocks. Analysis of Covariance (ANCOVA) models were used to determine the effect of time of day on the number of chickens that accessed the range and the frequency and duration of range visits, controlling for age, and included flock and time of day interaction. Post hoc analysis used the Bonferroni method to correct for multiple comparisons. Results are presented as raw means ± Standard Error (SE) unless otherwise noted.

## 3. Results

### 3.1. Range Availability

**Figure 2:**
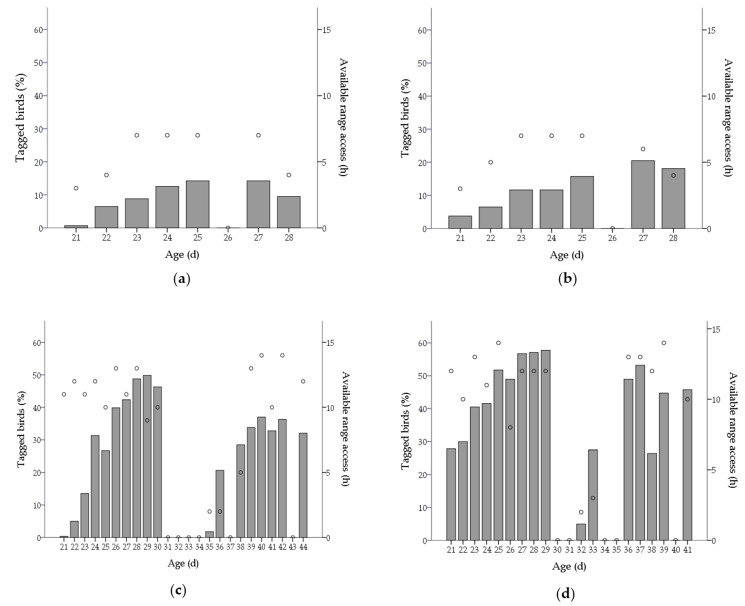
Bars indicate the proportion of chickens that accessed the range (%successfully tracked chickens; left y-axis) daily in winter (**a**) flock A (**b**) flock B and summer (**c**) flock C (**d**) flock D. Circles indicate time (hours; right y-axis) the range was available each day.

Due to weather extremes, management permitted range access seven out of 10 days prior to partial depopulation in winter flocks for a daily mean of 5.6 ± 0.4 h (total hours: flock A—37.5 h; flock B—37.8 h). Summer flocks had access to the range every day except 2 to 4 days prior to partial depopulation (flock C—10 days; flock D—9 days) and in total 18 and 16 days before complete depopulation for flocks C and D respectively; for a mean of 10.4 ± 0.6 h daily (total hours: flock C—183.8 h; flock D—168.7 h). Range access was not always provided continuously across days, predominantly due to adverse weather conditions. For winter flocks, there was one day of interruption on the sixth day after the range was first available due to adverse conditions (Figure 2). For summer flocks, there was two to four days of interruption immediately prior partial depopulation, and two to four intermittent days of interruption between partial depopulation and complete depopulation due to adverse weather conditions (Figure 2). Furthermore, range access was provided for only two hours the two days immediately after partial depopulation due to adverse weather conditions (Figure 2).

#### 3.1.1. Range Use

More than 93% of the 1200 tagged chickens were successfully tracked from day 21 until the end of the study (winter: flock A—98.3%, flock B—97.7%; summer: flock C—93.7%, flock D—94.7%), indicated by the recovery of functional tags at the end of trial. Figure 2 shows the percentage of the flock that accessed the range over time in summer and winter flocks. Fewer chickens accessed the range in the winter than in summer prior to partial depopulation (winter: 32.0 ± 0.8%, summer: 81.4 ± 6.0%; F_(1,3)_ = 68.0, *p* < 0.05). The total number of chickens that accessed the range was relatively similar within season replicates (winter: flock A—31.2%, flock B—32.8%; summer: flock C—75.4%, flock D—87.3%). Most chickens that accessed the range throughout the study did so prior to partial depopulation, in summer flocks (chickens that accessed the range for the first time before partial depopulation: flock C—92.5%, flock D—94.4% of ranging chickens).

The maximum number of chickens observed on the range at one time was 7.8% and 10.6% in winter, flocks A and B respectively (observed at 27 days of age in both flocks; 12:00 and 13:00 h, flocks A and B respectively) and 36.7% and 32.8% in summer, flocks C and D, respectively (observed at 29 days of age in flock C and 41 days of age in flock D, at 18:00 h in both flocks).

On average, ranging chickens accessed the range 34.4 ± 6.1% and 50.1 ± 1.4% of the available days up to partial depopulation (30–33 days of age) in winter and summer flocks, respectively. In summer flocks, ranging chickens accessed the range a mean of 43.5 ± 1.2% of the available ranging days up to complete depopulation (43–45 days of age). On each available ranging day before partial depopulation, chickens visited the range a mean of 3.2 ± 0.2 and 4.4 ± 0.1 times, for a mean of 7.9 ± 1.0 and 26.3 ± 0.8 min per visit, in winter and summer flocks respectively. After partial depopulation, chickens in summer flocks accessed the range a mean of 4.2 ± 0.1 times daily, for a mean of 23.4 ± 0.9 min per visit.

#### 3.1.2. Latency to Access the Range

The number of days that it took for a chicken to access the range for the first time, relative to the first day that range access was provided (hereafter referred to as “latency to access the range”) varied from the first available day of range access until the last day range access was provided (mean latency winter: flock A—3.9 ± 0.2 days, flock B—3.9 ± 0.2 days; mean latency summer: flock C—5.9 ± 0.2 days, flock D—3.7 ± 0.2 days). The number of chickens that accessed the range for the first time each day was not correlated with the cumulative number of days the range was previously available (hereafter referred to as “cumulative ranging day”) in winter flocks (flock A: *r*_(5)_ =−0.11, *p* > 0.05; flock B: *r*_(5)_ = −0.07, *p* > 0.05), but was negatively correlated with cumulative ranging day in summer flocks (flock C: *r*_(13)_ = −0.51, *p* = 0.05; flock D: *r*_(11)_ = −0.90, *p* < 0.001).

#### 3.1.3. Ranging Behaviour over Time

The two days immediately after partial depopulation in summer, flocks were excluded from the analysis as range access was provided ≤2 h due to adverse weather conditions. The number of chickens that accessed the range, total daily range visits, mean number of daily range visits/chicken and the mean duration of each range visit before partial depopulation in summer and winter flocks were all positively correlated with cumulative ranging day (*p* < 0.05), but not between partial and complete depopulation in summer (Table 1).

#### 3.1.4. Hours Available to Range

The number of hours the range was available for chickens varied between 3.0 to 7.2 h daily in winter flocks (mean 5.6 ± 0.4 h) and 2.0 to 14.0 h in summer flocks (mean 10.4 ± 0.6 h; Figure 2). The number of chickens on the range, total visits, total duration, and mean number of visits per chickens in both seasons were positively correlated with the number of hours the range was available (Table 2). The number of hours the range was available was positively correlated with the mean duration per visit in summer but not in winter (Table 2).

#### 3.1.5. Time of Day

Time of day had no effect on the number of chickens that accessed the range or the frequency, duration or mean duration per visit in winter flocks. However, range access was only provided between 11:00 h and 16:00 h most days in winter flocks.

In summer flocks, range access was provided inconsistently on some days due to adverse weather conditions (at the farm manager′s discretion). Thus, some data were excluded in the time of day analysis, only including data that was reflective of a “typical” ranging day. The criteria for exclusion included length of range access (days with < 2 h range access excluded), hours of range access (hours outside 09:00 and 20:00 were excluded) and day of range access (day one and two of range access (relative to first day range access was provided) were excluded due to unusually low levels of ranging).

**Figure 3:**
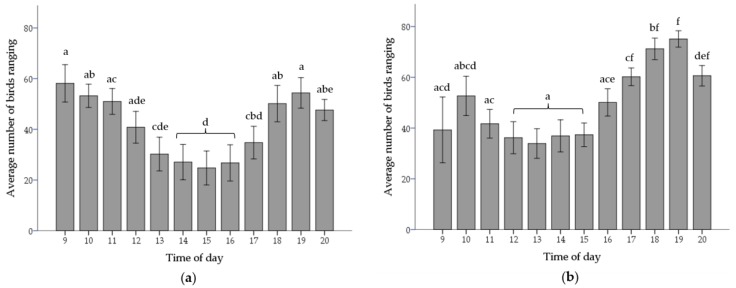
Mean number of chickens on the range (± Standard Error (SE)) during ranging hours (9:00 to 20:00 h) for summer flocks; flock C (**a**) and flock D (**b**).

There was an interaction between time of day and flock on the number of birds on the range (F_(11,290)_ = 2.12, *p* < 0.05). The number of chickens on the range and number of range visits peaked between 09:00 and 10:00 h and/or 18:00 and 19:00 h in summer flocks (number of chickens: Flock C—F_(11,149)_ = 2.79, *p* < 0.01; Flock D—F_(11,141)_ = 5.25, *p* < 0.001; Figure 3; number of visits: Flock C—F_(11,149)_ = 2.79, *p* < 0.01; Flock D—F_(11,141)_ = 5.10, *p* < 0.001). The mean duration of a range visit increased between 16:00 and 20:00 h in flock D (F_(11,141)_ = 9.76, *p* < 0.001) and peaked between 15:00 h and 16:00 h in flock C (F_(11,149)_ = 2.12, *p* < 0.05).

#### 3.1.6. Weather Conditions

As expected, weather conditions differed between seasons (Table 3).

For winter flocks, a multiple regression analysis was used to examine the relationships between the daily number of chickens on the range and outdoor environmental variables (Table 4). The model was significant (F_(3,13)_ = 23.24, *p* < 0.001) and accounted for 84.7% of variance in the number of chickens on the range. Rainfall significantly contributed to the model and accounted for 11.2% of the variation, indicating that higher daily rainfall was associated with less chickens on the range. The majority of the variance was explained by age (68.9%).

For summer flocks, a multiple regression analysis was used to examine the relationships between the number of chickens on the range hourly and both outdoor and indoor (shed) environmental variables (Table 4). The most parsimonious model accounted for 34.8% of the variance in the number of chickens on the range hourly (F_(6,357)_ = 32.74, *p* < 0.001). Indoor dew point was the greatest predictor of the number of chickens on the range; accounting for 10.9% of the variation and indicating that increased shed dew point was associated with fewer chickens on the range. Indoor temperature, age and flock contributed to the model; however, each accounted for less than 5% of the variance (Table 4).

#### 3.1.7. Pop-Hole Use

Although access to the range was provided via pop-holes evenly spaced along the shed, chickens in flocks A and C (seasonal replicates within shed 1) predominantly used two of the six available pop-holes (P2 and P3 in Figure 1a), which accounted for 47.8% of all range visits (F_(5,269)_ = 5.40, *p* < 0.05). These pop-holes were located at the front of the shed, adjacent to shade cloth on the range. However, these were not the only pop-holes with adjacent shade cloth. Chickens in flocks B and D (seasonal replicates within shed 2) predominantly used two pop-holes located at the front of the shed (P1 and P2 in Figure 1b; F_(6,313)_ = 6.50, *p* < 0.01), accounting for 41% of all range visits. The location of one of these pop-holes was directly adjacent to resources on the range (trees and shade cloth) but not the other. The number of range visits through a specific pop-hole did not differ according to whether a chicken was entering or exiting the range (shed 1: F_(1,269)_ = 0.10, *p* > 0.05; shed two: F_(1,313)_ = 0.20, *p* > 0.05).

#### 3.1.8. Turning the Litter

Data from the final day of ranging before partial depopulation in winter were excluded from all analysis as the farmer “turned the litter”. Throughout this day, 17.6% (*n* = 52) and 30.4% (*n* = 89) of the tracked chickens accessed the range; 38.5% (*n* = 20) and 43.8% (*n* = 39) of these chickens had not accessed the range prior to this day in flocks A and B, respectively.

## 4. Discussion

This study tracked individual broiler chickens on a commercial farm without segregating part of the shed, flock or range. Our results show that not all chickens accessed the outdoor range when given the opportunity. Chickens accessed the range on average three to four times for 1.5 to 2 h every two to three days for eight to 26 min per visit. Chickens did not immediately access the range when first given the opportunity, waiting an average of four days before accessing the range. The number of chickens on the range at one point in time was low, particularly in winter flocks, 7.8 to 10.6% in winter and 32.8 to 36.7% in summer, similar to previous studies using scan sampling methods [7,9,11,12,17]. However, the actual number of chickens that accessed the range over the course of the study was much higher; 31.2 to 32.8% in winter and 75.4 to 87.3% in summer, highlighting limitations in scan sampling method. Clearly, our understanding of commercial free-range broiler chicken ranging behaviour and implications for welfare will improve with advancement of technology. Currently, there is little technology that is affordable, reliable, and feasible for tracking an individual chicken’s precise location (indoor and outdoor) on commercial farms [18]. We found lower flock percentages of range use compared to previously reported RFID studies in Australia [12] and internationally [11], which may reflect differences in management, flock size, range design, strain (growth rate and length of time the range is available), or geographical differences including climate [5,7]. Segregating part of the flock may also have increased ranging behaviour in the previous RFID studies, given that the provision of vertical panels (e.g., fences) increases ranging behaviour in free-range laying hens [19]. Furthermore, the present study was conducted on larger flock sizes than previous studies. We provide evidence of factors that alter ranging behaviour including time and length of range exposure, shed design and shed environment.

The number of range visits and duration of range visits increased over time. Whether this is an effect of range exposure and familiarization and/or a reflection of age and development remains to be determined. The increased frequency of range visits with age we observed, in agreement with other studies [6,20], does not reflect broiler chicken age-related inactivity that has been previously reported in indoor and free-range housed broiler chickens [21,22]. However, we could not identify activity levels, and ranging visits may include time spent resting and lying down.

More than 90% of chickens that accessed the range in summer flocks did so prior to partial depopulation for slaughter. Furthermore, ranging behaviour (visits and duration) and the number of chickens on the range increased over the first two weeks until partial depopulation, but stabilised between partial depopulation and complete depopulation in summer flocks. Hence, we found no evidence that additional ranging opportunities provided beyond two weeks further increased ranging behaviour. In Australia, the typical length of time a fast-growing broiler chicken has to access an outdoor range is four weeks, given that range access is typically provided from 21 days of age, but it can be as little as 15 days if the individual is transported for slaughter at partial depopulation. We provide evidence that ranging opportunities prior to partial depopulation for slaughter are sufficient to establish ranging behaviour, relative to chickens that are permitted to range until complete depopulation.

Fewer chickens accessed the range in winter than summer, in agreement with previous studies [6,7]. Furthermore, they made fewer visits and spent less time on the range in winter, compared to summer. Ranging behaviour between flocks in the same season was relatively consistent, despite slight differences in flock size and range designs. Weather variables did not explain much of the variance in the number of chickens on the range in either season; rainfall in winter and shed dew point in summer had the greatest effect on ranging behaviour, each explaining less than 12% of the variance. Wind speed, rainfall, and indoor temperature each accounted for less than 5% of the variance in summer flocks. This may reflect the relatively few days of data collection and/or minimal variation within seasons, or that environmental conditions alone do not directly account for most of the variation observed between seasons.

Increased opportunities to range for summer flocks compared to winter flocks may explain differences in ranging behaviour between seasons. The provision of more ranging opportunities (both number of days and hours per day) was linked to a greater number of chickens on the range, visits to the range and time spent on the range. Relationships between increased opportunities and increased ranging behaviour (number of chickens and time spent on the range) have also been reported in laying hens [23].

As a consequence of shorter periods of ranging opportunities in winter flocks, the time of day when the range was available also differed between seasons and may partly explain variation in ranging behaviour. Summer flocks showed evidence of time of day effects on ranging behaviour displaying a diurnal ranging pattern in agreement with previous scan sampling studies [5,7]. Peak ranging times, including the number of chickens on the range and the number of range visits, occurred between 9:00 and 10:00 h and 18:00 and 19:00 h. Diurnal ranging patterns observed in summer flocks likely reflect diurnal rhythms. Broiler chicken foraging behaviour is typically displayed in diurnal peaks in the morning and evening and can be altered with changes in light intensity [24]. The range offers an ideal environment for foraging behaviours; indeed, foraging and ground pecking behaviours have been shown to be greater on the range compared to inside the shed [25]. Range access was rarely provided during these preferred times throughout winter. The typical pop-hole opening time in winter was between 11:00 and 12:00 h, closing between 16:00 and 17:00 h, compared to summer opening time 9:00 to 10:00 h, closing between 21:00 and 22:00 h. As such, it may be that broilers do not compensate by ranging at alternative times of the day when range access is not provided at favoured ranging times.

Evidence of range use to avoid negative stimuli was anecdotally observed in the current study; as the number of chickens on the range and the number of first time range users increased on the day the litter was turned in winter flocks. Turning the litter is often a critical management practice in commercial broiler sheds to maintain good litter quality and prevent associated effects on chicken welfare [26]. However, turning the litter may cause fear and stress, although controlled studies are lacking. The range area could offer an escape from this negative experience, although this was merely an observation on one day (in two sheds). Unfortunately, we did not track broiler chickens after this event and therefore do not know if chickens that accessed the range for the first time during litter turning would continue ranging on subsequent days.

Higher dew point and temperature inside the shed was predictive of fewer chickens on the range in summer flocks; whether this relationship was similar in winter remains unknown as we did not take these measures. This may be additional evidence that range use may be associated with avoiding negative stimuli such as sub-optimal shed conditions. However, causation cannot be inferred in this study, and it is possible that these findings indicate the effect of chickens on the shed environment, through less chickens ranging, hence higher shed stocking density and consequently higher metabolic heat production raising indoor shed temperature and dew point, rather than environmental conditions in the shed encouraging chickens to range. These results do highlight the importance of monitoring the shed environment in relation to ranging behaviour and considering that range access may be related to negative stimuli rather than associated with a positive aspect of the range environment. The influence of the indoor environment is an aspect often overlooked in ranging studies.

We observed a flock preference for specific pop-holes in both sheds (P2 and P3 in shed one, and P1 and P2 in shed two; Figure 1). We could not identify the characteristics of preferred pop-hole location and design; this could be related to areas with human disturbance, brooding areas, location of noisy fans at the rear of the shed or protection from weather extremes (wind or UV light). The preferred pop-holes did not appear related to resources on the range such as trees or shade cloths, despite range resources often being the focus of studies of ranging preferences [7,8,27]. Two of the favoured pop-holes were directly under shade cloths in shed one, but not in shed two, and not all pop-holes with adjacent shade cloth on the range were favoured in either shed. Characteristics of favoured pop-holes may consequently affect range use and should be investigated further to optimise transition from the shed environment to the range area.

This study provides knowledge regarding ranging behaviour in commercial free-range broiler chickens in relation to age and management and environment variability. Whilst obtaining data on commercial farms have numerous benefits, there are limitations. We make the assumption that the tracked chickens in each flock are representative of ranging behaviour in the whole flock, as careful sampling methods when choosing focal chickens to tag should theoretically provide a representative subsample of the population. Of greater importance, the results were obtained from one farm in one region of Australia, and means of ranging behaviour may not be representative of the most extensive rangers in the flock (see paper two in this series). Furthermore, our study could not assess how far chickens ranged, what range locations are favoured, or activity levels in the shed and range areas. This study was conducted on one strain of broiler chicken that is typically housed in Australian commercial free-range production systems (ROSS 308). Results may differ with slower growing strains used in other countries for free-range production (e.g., ROSS 708 or other strains). As there are few scientific investigations regarding ranging behaviour in broiler chickens, this study provides important knowledge to direct further investigations into the factors affecting ranging behaviour.

## 5. Conclusions

This study is the first to monitor individual ranging behaviour of free-range broiler chickens on a commercial farm without altering flock size or shed environment. Tracking chickens through RFID revealed higher estimates of ranging behaviour than previous studies using scan sampling methods. Ranging behaviour increased from first day of range access for two weeks and stabilised thereafter in summer flocks. Fewer chickens accessed the range in winter flocks than summer flocks. Chickens that did range in winter flocks did so less frequently and for a shorter period of time compared to ranging chickens in summer flocks. However, ranging behaviour was relatively consistent within each season. We found little evidence that seasonal differences in ranging behaviour were solely or directly related to variation in weather. Differences in ranging behaviour between seasons may also be due to reduced ranging opportunities in winter (number of days the range was available and length of time) and the time of day the range was available, although these factors are often inherently linked to weather conditions permitting ranging. This study highlights the importance of obtaining a detailed understanding of the influence of range and shed design and environmental and management factors to provide commercial broiler chickens with optimal conditions to range.

## Figures and Tables

**Table 1:** Pearson’s partial correlation coefficients (*r*), controlling for flock, between the cumulative ranging day (relative to first availability) and daily ranging behaviour, before partial depopulation (summer and winter flocks) and between partial and complete depopulation (summer flocks only).

Daily Ranging Behaviour	Cumulative Ranging Day (from First Access to Partial Depopulation)		Cumulative Ranging Day (from Partial Depopulation to Complete Depopulation)
	Winter (*n* = 14 days)	Summer (*n* = 19 days)	Summer (*n* = 11 days)
Number of chickens that accessed the range	0.86 ***	0.94 ***	0.22
Total daily range visits	0.79 ***	0.76 ***	0.06
Mean daily visits/individual	0.65 *	0.53 *	0.03
Mean duration/visit	0.76 ***	0.79 ***	0.41

Note: * and *** indicates significance at *p* < 0.05 and 0.001 levels respectively.

**Table 2:** Pearson’s partial correlation coefficients (*r*), controlling for flock and age, between the number hours the range was available each day and ranging behaviour for winter and summer flocks.

Ranging Behaviour	Range Access (Hours/Day)	
	Winter (*n* = 14)	Summer (*n* = 34)
Number of chickens that accessed the range	0.69 **	0.55 ***
Total daily range visits	0.74 ***	0.57 ***
Mean daily visits/individual	0.85 ***	0.62 ***
Mean duration/visit	−0.06	0.53 ***

Note: ** and *** indicates significance at the 0.01 and 0.001 level respectively.

**Table 3:** Mean daily (± Standard Error (SE)) environmental conditions in winter (*n* = 8 days) and summer (*n* = 26 days). Environmental conditions were measured twice daily in winter and at ten minute intervals in summer.

Variable	Winter	Summer
Minimum outdoor temperature (°C)	3.6 ± 1.2	11.1 ± 0.7
Maximum outdoor temperature (°C)	12.6 ± 0.6	29.9 ± 1.1
Minimum indoor shed temperature (°C)	19.5 ± 0.3	17.8 ± 0.2
Maximum indoor shed temperature (°C)	22.2 ± 0.4	28.5 ± 0.5
Outdoor relative humidity (%)	80.6 ± 3.1	63.9 ± 2.0
Indoor shed relative humidity (%)	68.4 ± 0.3	64.0 ± 0.4
Indoor shed dew point (°C)	–	15.37 ± 0.1
Daily rain fall (mm)	3.4 ± 2.0	1.8 ± 1.3
Wind speed (km/h)	12.2 ± 1.9	4.2 ± 0.6
Ultraviolet radiation (uW/cm^2^)	–	853.5 ± 47.4
Sunrise (h)	07:29–07:33	05:51–05:58
Sunset (h)	17:07–17:08	20:29–20:44

**Table 4:** Multiple regression analysis on the number of chickens on the range daily in winter, and hourly in summer (adjusted r^2^ = 0.76 and 0.35 in winter and summer, respectively). Only variables that significantly contributed to the most parsimonious model are presented.

Predictor	Beta Coefficient (Standardised)	*t*_(5, 360)_	Partial Correlation Coefficient
**Winter**			
Rainfall	−0.34 *	−2.46	−0.34
Age	0.83 **	6.06	0.83
**Summer**			
Indoor dew point	−0.44 **	−8.35	−0.36
Rainfall (daily)	−0.26 **	−4.80	−0.21
Indoor temperature	−0.22 **	−4.19	−0.18
Wind speed	0.12 **	2.70	0.12
Age	0.22 **	4.61	0.20
Flock	0.18 **	4.61	0.20

* and ** indicates significance at the 0.05 and 0.01 level, respectively.
